# The High Cost of HIV-Positive Inpatient Care at an Urban Hospital in Johannesburg, South Africa

**DOI:** 10.1371/journal.pone.0148546

**Published:** 2016-02-17

**Authors:** Lawrence C. Long, Matthew P. Fox, Celeste Sauls, Denise Evans, Ian Sanne, Sydney B. Rosen

**Affiliations:** 1 Department of Internal Medical, School of Clinical Medicine, Faculty of Health Sciences, University of Witwatersrand, Johannesburg, South Africa; 2 School of Public Health, Faculty of Health Sciences, University of Witwatersrand, Johannesburg, South Africa; 3 Health Economics and Epidemiology Research Office, Wits Health Consortium, Johannesburg, South Africa; 4 Center for Global Health & Development, Boston University, Boston, Massachusetts, United States of America; 5 Department of Epidemiology, Boston University School of Public Health, Boston, Massachusetts, United States of America; Medical University of Vienna, AUSTRIA

## Abstract

**Background:**

While most HIV care is provided on an outpatient basis, hospitals continue to treat serious HIV-related admissions, which is relatively resource-intensive and expensive. This study reports the primary reasons for HIV-related admission at a regional, urban hospital in Johannesburg, South Africa and estimates the associated lengths of stay and costs.

**Methods and Findings:**

A retrospective cohort study of adult, medical admissions was conducted. Each admission was assigned a reason for admission and an outcome. The length of stay was calculated for all patients (N = 1,041) and for HIV-positive patients (n = 469), actual utilization and associated costs were also estimated. Just under half were known to be HIV-positive admissions. Deaths and transfers were proportionately higher amongst HIV-positive admissions compared to HIV-negative and unknown. The three most common reasons for admission were tuberculosis and other mycobacterial infections (18%, n = 187), cardiovascular disorders (12%, n = 127) and bacterial infections (12%, n = 121). The study sample utilized a total of 7,733 bed days of those, 55% (4,259/7,733) were for HIV-positive patients. The average cost per admission amongst confirmed HIV-positive patients, which was an average of 9.3 days in length, was $1,783 (United States Dollars).

**Conclusions:**

Even in the era of large-scale antiretroviral treatment, inpatient facilities in South Africa shoulder a significant HIV burden. The majority of this burden is related to patients not on ART (298/469, 64%), and accounts for more than half of all inpatient resources. Reducing the costs of inpatient care is thus another important benefit of expanding access to ART, promoting earlier ART initiation, and achieving rates of ART retention and adherence.

## Introduction

Although widespread access to antiretroviral therapy (ART) is commonly assumed to have reduced the need for HIV-related hospital inpatient care, there is little published evidence confirming this in low- and middle-income countries. In South Africa, where an estimated 6.3 million people are HIV-infected and more than 2.6 million are on ART [1], the national HIV treatment program places a significant burden on the public healthcare system. While most HIV care is provided on an outpatient basis at public primary health clinics, hospitals continue to treat serious HIV-related admissions but the current extent and cost of this is unknown. Compared to outpatient care, inpatient care is resource-intensive and expensive. It is thus important to describe the main reasons for HIV-related admissions and the associated costs.

Before the availability of public HIV treatment in South Africa, inpatient facilities saw an increasing number of HIV patients presenting with serious HIV-related complications and AIDS [2–5]. South Africa launched large-scale, public HIV treatment in 2004 through outpatient facilities, and most recent studies of the costs of HIV care in South Africa have been limited to outpatient services [6–9]. Studies estimating inpatient costs have typically included them as part of the overall cost of HIV treatment [10–16]. Only two have looked specifically at inpatient costs [17, 18], and both relied on data from the earliest years of the ART rollout. Similarly, only a few published studies have examined the share of overall inpatient resource utilization attributable to HIV since the advent of public sector ART in 2004 [17, 19, 20], and none have estimated this in more recent years. As a result, the current extent of HIV-related inpatient admissions and its associated cost in public sector hospitals is not known.

To provide current information to policy makers and program managers about the burden of HIV-related inpatient care in a setting of widespread ART access, this study reports the primary reasons for HIV-related admission at a regional, urban hospital in South Africa during 2010 by HIV and ART status and estimates the associated lengths of stay and costs. Data are drawn from medical records and hospital invoices, providing more detailed and accurate information than has been available thus far.

## Methods

### Study Population and Data

We conducted a retrospective cohort study of admissions at a regional, urban, public sector hospital in Johannesburg, South Africa. The hospital, with 11 inpatient medical wards and 347 beds, falls under the Gauteng Province Department of Health and serves a large urban catchment area that includes both formal and informal settlements.

The study population included all adult (≥18 years old) admissions that occurred at the medical admission wards between January 1 and August 31, 2010. This time period (mid summer to late winter) was selected to capture seasonal variation in inpatient medical admissions. Admissions into other specialist wards (e.g. gynecology) were excluded. We created a sampling frame from the medical admission ward registers, then drew a random 12% sample of admissions for analysis, with an aim of 10% and an additional 2% selected to compensate for anticipated missing files. Repeat admissions of the same individual patient were recorded as two separate admissions.

Data were collected from three sources, reflecting the hospital’s record keeping system. Medical admission ward registers provided the date of admission, preliminary diagnosis, and final ward assignment. Patient demographic characteristics and all the care that the patient received during the admission were drawn from inpatient files. Finally, the electronic laboratory system was used to verify and update diagnostic tests done during the admission. Final data collection for a patient occurred at least 6 months after admission to ensure sufficient time for the admission to be completed and the patient file stored. Up to three attempts were made to locate missing inpatient admission files over a 3-month period. If the files could still not be located, the admissions were classified as missing. Missing or otherwise incomplete files were excluded.

Patient data were extracted from the inpatient files and entered directly into a CS Pro^™^ database. For patients known to be HIV-negative or of unknown HIV status, only demographic and basic admission data were extracted. For HIV-positive patients, we also extracted detailed resource usage data, including length of stay, drugs prescribed, intravenous fluids prescribed, diagnostics performed, and any other recorded procedures.

### Outcomes and HIV Status

For each admission in the sample, we started by assigning a reason for admission, corresponding to the final discharge diagnosis. Final discharge diagnosis was extracted from the paper patient discharge summary. If the discharge summary was incomplete or missing then the patient medical notes were reviewed to see if a final discharge diagnosis was noted. In the absence of a clear discharge diagnosis the diagnosis was classified as “Unknown”. Each admission was assigned one of four admission outcomes: transfer, unknown, died, discharged. Transfers were those patients sent to other facilities for additional treatment. Patients with unknown outcomes did not have a date of discharge, death, or transfer; their data is included in the baseline cohort description and analysis of reasons for admission but not in average length of stay or cost estimates. Deaths and discharges were as recorded in the inpatient record.

We then categorized each enrolled subject into one of three groups. ‘*HIV-positive’* contained known HIV-positive patients determined by a conclusive result in the inpatient file, the presence of HIV monitoring diagnostics, and/or ARV prescriptions. This categorization does not specify whether or not the reason for admission was HIV related, rather the status of the person being admitted. ‘*HIV-negative’* included patients with known HIV-negative status in their admission records. ‘*HIV unknown’* encompassed all other patients, including undiagnosed HIV-positive patients for whom HIV status was not recorded in the inpatient file. We report demographic characteristics and primary reason for admission, categorized by major disease area, by HIV status.

### Resource Utilization and Costs

For inpatient care, a substantial portion of total admission cost is attributable not to variable resources, such as drugs, diagnostics, and procedures, but rather to the resources associated with the stay itself, often called the “hotel” costs and including the hospital infrastructure, general staffing, and so on. For each HIV status group and primary reason for admission, we first estimated and compared the mean length of stay and hotel cost associated with it, with 95% confidence intervals.

For HIV-positive patients, we also estimated actual utilization and associated costs for variable resources as well as hotel costs, and we report these admission costs by cost category with means and proportions. Costs were measured from the provider (hospital) perspective and included the cost of all resources from the date of admission of an HIV-positive patient to the date of outcome (i.e. discharge, death, transfer out). A bottom up costing approach was used, including drugs, fluids, diagnostics, surgical procedures and hotel costs (including bed cost, support staff, and medical staff). Bed cost included equipment, space, shared staff, administration and overhead at the study site. Unit costs of drugs, diagnostics, lab charges, procedures, and other variable inputs were recorded from the most recent available invoices / price lists. All unit costs were in 2013 South African Rand (ZAR) or adjusted to 2013.

For medical staff costs, the complement of medical staff in the medical wards was estimated during the study period and 2013 government salary costs applied [21]. Since doctors do not spend all their time in the medical wards, we allocated 20%, 40% and 60% of specialist/consultant, registrar, and medical officer time to the medical wards, respectively. Nurses were assumed to spend all their time in the medical wards. Total personnel costs were then divided by the number of annual bed days to estimate the medical staff cost per day of medical admission.

Annual costs of inpatient care (including all hotel and standard care costs but excluding variable costs for condition-specific drugs, diagnostics, and procedures and medical staff costs) were taken from the hospital’s annual accounts for the relevant year (2012–2013) and apportioned either by percentage of medical beds to overall hospital beds or by physical space in the medical wards to overall space in the hospital. Equipment in the medical wards was inventoried, unit costs applied, and an annual equivalent cost calculated using a working life of 8 years and a discount rate of 5%. For all other shared space, equipment was estimated at 20% of the cost of the space, the same percentage as in the medical ward. Space costs were estimated by obtaining minimum rental costs per square metre from the surrounding area at 2013 prices. The hotel, equipment, and space costs are reported together as fixed costs. The reported costs were converted from South African Rand (ZAR) to United States Dollar (USD) at the average exchange rate prevailing during 2013, which was 9.6:1 (ZAR:USD) [22]. Note that costs included may be paid by any combination of patient fees, government budgets, donor grants, and other sources of funding.

The study protocol was reviewed by the Institutional Review Boards of the University of Witwatersrand and Boston University, who approved the collection of the data without informed consent and the use of an anonymous analytic dataset.

## Results

During the eight-month study period there were 10,801 admissions to the medical wards. A random sample of 1,343 (12%) was selected; 302 (2.8%) were then excluded because their files were missing or incomplete, leaving an analytic sample of 9.2% of all admissions. The excluded admissions were matched to the hospital death register and the electronic laboratory records and did not differ from the analytic sample, suggesting that the excluded admissions were not sicker or more likely to be HIV positive than those included. The remaining 1,041 constituted the analytic dataset, with just under half (45%, n = 469) of the sample having a confirmed HIV-positive diagnosis. (See S1 Fig)

The baseline characteristics of the sample are shown in Table 1. Just under half were known to be HIV-positive patients; the rest were HIV-negative (20%) or of unknown HIV status (35%). Almost two thirds (59%) of patients admitted were between 25–50 years of age; amongst HIV-positive patients more than four out of five (82%) were in this age range, consistent with national HIV prevalence by age group. Patients with unknown HIV status were substantially older than those with a known HIV status, suggesting that older patients are less likely to have been tested for HIV. For the HIV-positive patients with CD4 counts (n = 399/469), 36% were at very high risk for HIV-related illness (CD4 count < 50 cells/mm^3^) and only 37% were recorded as being on treatment. The percentages of deaths (17%) and transfers (2%) were higher amongst HIV-positive admissions compared to HIV-negative (6% and 0%) and unknown (9% and 1%). Unknown outcomes were evenly distributed by HIV status.

**Table 1:** Baseline characteristics of admission cohort.

	Overall n = 1041		HIV status					
			Positive n = 469		Negative n = 206		Unknown n = 366	
**Gender, n (%)**								
Male	486	(46.7)	216	(46.1)	114	(55.3)	156	(42.6)
Female	555	(53.3)	253	(53.9)	92	(44.7)	210	(57.4)
**Age, n (%)**								
18–24	65	(6.2)	16	(3.4)	28	(13.6)	21	(5.7)
25–30	139	(13.4)	81	(17.3)	27	(13.1)	31	(8.5)
31–40	281	(27.0)	194	(41.4)	41	(19.9)	46	(12.6)
41–50	199	(19.1)	113	(24.1)	42	(20.4)	44	(12.0)
51–60	156	(15.0)	44	(9.4)	29	(14.1)	83	(22.7)
61–70	102	(9.8)	21	(4.5)	22	(10.7)	59	(16.1)
>70	99	(9.5)	0	0	17	(8.3)	82	(22.4)
**Age, median (IQR)**	42	(32, 56)	37	(32, 45)	42	(29, 55)	55	(39, 68)
Most recent* **CD4 count (cells/mm**^**3**^**), n (%)**								
Missing	-	-	70	(14.9)	-	-	-	-
< = 50	-	-	142	(30.3)	-	-	-	-
51–100	-	-	63	(13.4)	-	-	-	-
101–200	-	-	79	(16.8)	-	-	-	-
201–350	-	-	65	(13.9)	-	-	-	-
>350	-	-	50	(10.7)	-	-	-	-
Most recent* **CD4 count (cells/mm**^**3**^**), median (IQR)**	-	-	90	(30, 237)	-	-	-	-
**ARV Status, n (%)**								
Not on ARVs	870	(83.6)	298	(63.5)	206	(100.0)	366	(100.0)
On ARVs	171	(16.4)	171	(36.5)	-	-	-	-
**Outcome, n (%)**								
Discharged	876	(84.1)	368	(78.5)	188	(91.3)	320	(87.4)
Died	126	(12.1)	80	(17.1)	13	(6.3)	33	(9.0)
Transferred	13	(1.2)	11	(2.3)	-	-	2	(0.5)
Unknown	26	(2.5)	10	(2.1)	5	(2.4)	11	(3.0)

* Done during or within the 3 months prior to admission

### Reason for admission

**Fig 1:**
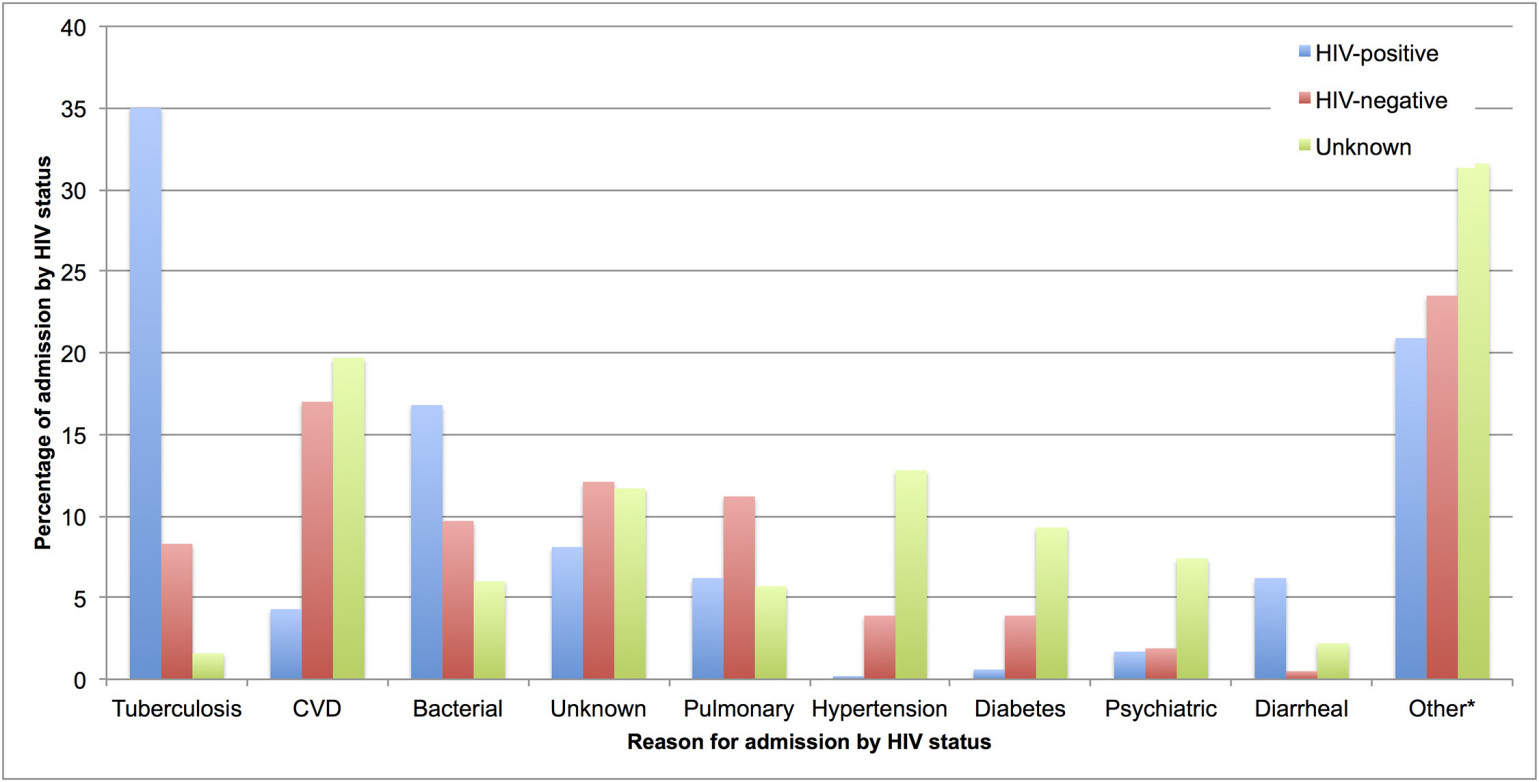
Summary of reasons for admission by HIV status. This figure presents the top 5 reasons for admission for each HIV status category. All other reasons were collapsed into the other category.* Tuberculosis was the most prevalent reason for admission amongst HIV-positive admissions accounting for 35%. CVD was the single most prevalent reason for admission amongst HIV-negative admissions accounting for 17%, while it only accounted for 4% of all HIV-positive admissions.

There were 326 unique reasons for admission, which were categorized into 25 major disease areas. (See Table A in S1 File.) The top 5 reasons for admission for each HIV status resulted in 9 unique reasons and accounted for more than 75% of all admissions. Fig 1 presents these results by reason for admission. (See Table B in S1 File)

The three most common reasons for admission were tuberculosis and other mycobacterial infections (18%, n = 187), cardiovascular disorders (12%, n = 127) and bacterial infections (12%, n = 121). The majority of tuberculosis (87%) and bacterial infections (65%) were found among HIV-positive patients, while the majority of cardiovascular disorders (57%) were reported amongst those with unknown HIV status. Pulmonary disease was proportionately almost double in the HIV status unknown patients, which may be explained by the higher median age of this group. Non-communicable diseases such as hypertension, diabetes, and psychiatric illness were much more common amongst those with an unknown HIV status compared to either HIV-positive or negative patients. The reason for admission for 10% (n = 106) of admissions was unknown. This was either because the information was missing from the patient record or the attending physician did not record a conclusive discharge diagnosis.

We also stratified reason for admission by ART status and baseline CD4 count for those with HIV. (See Table C in S1 File.) Although most (63%) HIV-positive admissions were not on ART, the 5 most common reasons for admission were the same regardless of treatment status, with the exception of bacterial infection, which comprised a smaller share of admissions among patients on ARVs (9.4%) than not (21.1%) (prevalence difference 11.7%; 95% CI 5%, 18%).

**Fig 2:**
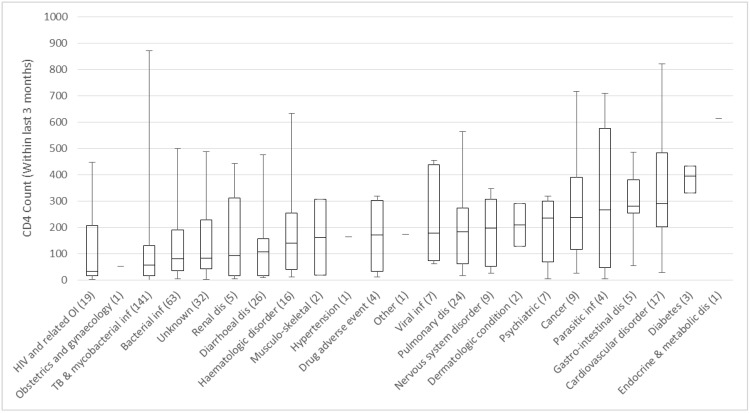
Box and whisker plot of CD4 count by reason for admission. The distribution of CD4 count at admission shows that all HIV positive admissions with one exception had a median CD4 count below 500 cells/mm^3^. This suggests that boosting the CD4 count of HIV positive patients by starting treatment early and ensuring adherence while on treatment may reduce the frequency and severity of admission.

As Fig 2 indicates, the top 5 reasons for HIV-positive admissions all had a median CD4 count below 200 cells/mm^3^, which was the threshold for ART initiation at the time of the study. As might be expected, TB and other mycobacterial infections, which account for a major portion of HIV-related inpatient admissions, become less frequent as baseline CD4 count rises. Nearly half (48%) of admissions among patients with CD4 counts < = 50 cells/mm^3^ were attributable to TB and mycobacterial infections (51–100 cells/mm^3^: 38%, 101–200 cells/mm^3^: 34%, 201–350 cells/mm^3^: 23%, >350 cells/mm^3^: 14%).

### Length of stay and distribution of bed days

Among all admissions with a known outcome (n = 1,015), the overall mean length of stay (95% CI) was 7.6 days (7.2, 8.0). The longest average length of stay was for HIV and related OIs at 13.2 days (8.9, 17.3). HIV-positive admissions had a longer average length of stay overall of 9.3 days, compared to 7.3 and 5.6 days for HIV-negative and HIV unknowns. Among HIV-positive patients, renal disease (18 days), gastro-intestinal disease (15 days), and drug adverse events (14 days) resulted in the longest average stays. For HIV-negative and HIV unknown admissions, cancer resulted in the longest average stay (23 and 10 days respectively). (Full details in Tables D and E in S1 File)

The admissions included in the study sample utilized a total of 7,733 bed days and the top 5 contributors to bed days by HIV status are included in Table 2. (Full details in Table F in S1 File) Of those, 55% (4,259/7,733) were for HIV-positive patients, 19% (1,469/7,733) were not related to HIV, and 26% (2,005/7,733) were for patients of unknown HIV status. TB and other mycobacterial infections accounted for the most (18%) bed days, driven largely by the HIV-positive admissions of which 36% were as a result of TB and other mycobacterial infections. Cardiovascular disease was the leading reason for bed day usage amongst HIV-negative and HIV unknown patients, accounting for 17% and 20% respectively.

**Table 2:** Distribution of bed days by HIV status and reason for admission.

Reason for admission	Overall		HIV status					
			Positive		Negative		Unknown	
Total bed days (%)								
TB and other mycobacterial infections	1852	(18.3)	1617	(35.5)	201	(8.5)	34	(1.7)
Cardiovascular disorder	936	(12.5)	197	(4.4)	249	(17.4)	490	(20.3)
Bacterial infection	887	(11.9)	625	(17.2)	136	(10.0)	126	(6.2)
Unknown reason for admission	687	(8.0)	356	(6.3)	131	(10.0)	200	(9.0)
Pulmonary disease	480	(7.2)	207	(6.3)	151	(11.4)	122	(5.9)
Hypertension	273	(5.5)	1	(0.2)	47	(4.0)	225	(13.2)
Diabetes	253	(4.4)	27	(0.7)	46	(4.0)	180	(9.6)
Diarrheal disease	205	(3.7)	175	(6.3)	2	(0.5)	28	(2.3)
Psychiatric	180	(3.8)	57	(1.7)	28	(2.0)	95	(7.6)
Other*	1980	(24.5)	997	(21.4)	478	(32.3)	505	(24.2)
Total**	***7733***	***(100*.*0)***	***4259***	***(100*.*0)***	***1469***	***(100*.*0)***	***2005***	***(100*.*0)***

* Includes all other reasons which were not amongst the top 5 highest contributors towards total bed days.

** All admissions where the outcome is unknown are excluded, as they do not have a discharge date.

### Cost of HIV-positive admissions

Table 3 presents the top ten most expensive reasons for admission for the HIV-positive patients. (Full details in Table G in S1 File) The average cost per admission amongst confirmed HIV-positive patients, which was an average of 9.3 days in length, was $1,783. This resulted in an average cost per bed day of approximately $192, which is just slightly less than the patient day equivalent (PDE) cost reported for Gauteng Province hospitals of $225 [23]. The majority of the cost can be attributable to medical staff (42%) and fixed costs (35%). Laboratory and drugs costs accounted for only 11% and 3% respectively. Although 36% of all HIV-related admissions were reported to be on antiretrovirals, almost none were dispensed during the hospital admission, suggesting that either patients brought their ARVs with them or treatment was interrupted during the hospital stay.

**Table 3:** Cost for HIV-positive admissions (HIV-positive only), by cost category in United States Dollars (USD).

Reason for admission	N	All admissions (USD)		Mean per admission (USD)					
		Total	(%)	Fixed*	Medical staff	Lab	Drug & ARV	Fluid	Total
Renal disease	6	23,661	(2.9)	1,241	1,459	372	110	762	3,944
Drug adverse event	5	12,881	(1.6)	961	1,129	234	21	232	2,576
Endocrine and metabolic disease	1	2,472	(0.3)	905	1,063	363	140	1	2,472
HIV and related OI	21	51,141	(6.3)	918	1,079	218	83	137	2,435
Gastro-intestinal disease	5	11,972	(1.5)	1,030	1,211	132	20	2	2,394
Unknown reason for admission	29	68,150	(8.3)	652	766	219	69	198	2,350
Hematologic disorder	16	32,612	(4.0)	648	762	241	52	336	2,038
Parasitic infection	5	9,637	(1.2)	696	818	189	98	126	1,927
TB and other mycobacterial infections	163	313,318	(38.3)	686	806	198	66	165	1,922
Musculo-skeletal	2	3,761	(0.5)	800	941	93	44	3	1,880
Other	206	288,553	(35.3)	515	605	162	52	67	1,401
***All***	***459***	***818*,*156***	***(100*.*0)***	***632***	***743***	***188***	***61***	***134***	***1*,*783***

* Fixed costs include hotel, equipment and space costs.

Renal disease generated the most expensive admission ($3,944 per admission, 17.8 days) driven by the length of stay, but this was relatively infrequent (6/459). TB and other mycobacterial infections are ranked ninth by average cost per admission but occurred much more frequently (163/459). As a result TB accounts for the largest portion of HIV-related admission costs, more than a third of the total.

## Discussion

In a random sample of 1,041 inpatient admissions at a regional hospital in Johannesburg in 2010, nearly half (45%) were for patients known to be HIV-positive. These patients accounted for at least 55% of all resources utilized (bed days) by the sampled admissions. Since some proportion of the admissions for patients of unknown HIV status were bound to have been for HIV-positive patients, these figures represent the minimum burden placed on the hospital by HIV-positive patients during the time of the study. They are comparable to the results of a 2005 study of another Johannesburg hospital [17], suggesting that the burden of HIV on South African hospitals may not have changed substantially since the rollout of ART in 2004.

Only 36% of HIV-positive patients were reported to be on ART at the time of the admission, despite 71% being eligible for ART based on their CD4 count. A recent report [18] on an HIV-positive cohort in South Africa found that the majority (64%) of hospitalizations occurred prior to the initiation of ART, which may explain the findings of this paper. In our study, only 12% of HIV-positive admissions occurred when the CD4 count > 350 cells/mm^3^. Earlier treatment initiation, at the higher CD4 count thresholds adopted by the South African Government in 2011 and again in 2015, may therefore substantially reduce the number of HIV-related admissions in future years.

In our sample, tuberculosis accounted for almost a fifth of all inpatient medical resources used. Given the low median CD4 count at admission, it is not surprising that tuberculosis and other similar infections were the leading reason for admission and death of HIV-positive patients causing one fourth of all HIV-related deaths. Similar results have been seen in other studies both in sub Saharan Africa prior to treatment availability [24–26] and in South Africa soon after treatment was made available [17–19]. Cardiovascular disease was the leading reason for admission amongst those who had a known HIV-negative or unknown status, who were also older than those with HIV. This profile is more typical of areas or countries where the population is not severely immune compromised. The UK reported ischemic heart disease as the third primary reason for admission after cancer and pneumonia [27]. The US reported congestive heart disease as the fourth primary reason for admission after live births, pneumonia and osteoarthritis [28].

Within the HIV-positive patients, reason for admission did not vary by ART status. The CD4 count profile for HIV-positive patients by treatment status was also similar, which could account for the similar admission profile and also could suggest that those on ART are recent initiates. Leisegang and colleagues followed HIV-positive patients both prior to and after ART initiation and showed that the highest inpatient costs occurred in the period immediately prior to, during and after ART initiation [15].

A number of papers have indicated that it is the non-curative costs (i.e. hotel costs, not drugs and diagnostics) which drive the cost of medical admissions, meaning that length of stay is an accurate proxy for cost of admission [17, 19]. Overall HIV-positive patients had longer average medical admissions compared to other patients and as a result could on average be expected to use more resources and cost more per admission than HIV-negative patients. Renal disease and cancer had the longest average medical admissions amongst the HIV-positive and negative patients, respectively.

The cost of HIV-related admissions to the South African healthcare system is not trivial, either in terms of the facility resources it takes from other potential patients nor in financial terms. Another recent study reported that for South African patients on ART, the hospitalization rate was 6.9 admissions per 100 patient years [18]. With over 2 million patients on ART in South Africa, this suggests that there are some 138,000 medical admissions in the country per year just for patients already on ART. We estimated an average cost of $1,783 per admission, bringing the annual cost of ART medical admissions to $246 million. With a year of ART estimated to cost approximately $404 [29], the annual funds spent on HIV inpatient admissions while on ART could pay for the annual cost of over 600,000 patients on ART, more than a quarter of the patients on treatment in 2013 [1].

If South African inpatient healthcare facilities are operating at capacity, then a high HIV inpatient burden may lead to the crowding out or displacement of patients with other conditions [26]. The Gauteng HIV prevalence in 2012 for adults (15–49 years old) was 17.8% [30], yet in our sample, HIV positive patients utilized 55% of inpatient resources. In other words, less than a fifth of the population consumed more than half of the available resources, creating a large excess burden of HIV on inpatient facilities. Diminishing this excess burden through earlier ART initiation and better medication adherence—high priorities of the South African government—may free up additional capacity and reduce the cost of treating HIV patients, adding further evidence of the importance of these goals.

Our study had a number of limitations. Other research has suggested that specialist wards may also experience a large HIV burden [31], but this study focused on medical ward admissions only. The retrospective nature of the study meant that we relied on routine hospital files and a number of these were missing or incomplete. In order to minimize the impact of this missing information alternative data sources (hospital death register, electronic laboratory records) were used to provide additional information on these patients. While we believe the length of stay to be accurate, the estimated costs are based on resource usage extracted from medical records, which may not report all resources utilized. Given that the majority of costs are driven by length of stay, missed variable resources are unlikely to have a major effect on results, however.

## Conclusion

Even in the era of large-scale antiretroviral treatment rollout, we found that inpatient facilities in South Africa are still shouldering a significant HIV burden. We found that the majority of the burden of HIV inpatient care on hospitals is related to patients not on ART, and that this burden is large, accounting for more than half of all inpatient resources. Reducing the costs of inpatient care is thus another important benefit of expanding access to ART, promoting earlier ART initiation, and achieving rates of ART retention and adherence. Having regular reports on HIV-related hospital admissions would provide critical insight into the success of the national treatment program in reducing both HIV-related morbidity and healthcare costs.

## Supporting Information

S1 Fig Cohort enrolment. There were 10,801 medical admissions between January and August 2010. A sample of 10% was targeted with an additional 2% to account for missing or incomplete files. Of the 1,343 files selected 220 could not be found and an additional 82 could be identified but the admission of interest was missing or incomplete in the file. There were 1,041 admissions captured representing 9.6% of all admissions during the period. The majority of the admissions were confirmed as HIV-positive (466, 45%), while the rest were HIV-negative (206, 20%) and those with an unknown HIV status (366, 35%).
(TIFF)

S1 File Table A. Reason of admission by HIV status. Table B. Summary of reasons for admission by HIV status. Table C. Primary reason for admission by ART status, HIV-positive only. Table D. Length of stay (days) by reason of admission and HIV status. Table E. Summary length of stay (days) by HIV status and reason for admission. Table F. Distribution of bed days by reason of admission. Table G. Mean cost per admission (HIV-positive only), by cost category in USD.
(DOCX)
