# Supplementary figures and images for: The High Cost of HIV-Positive Inpatient Care at an Urban Hospital in Johannesburg, South Africa
Source: PLoS One. 2016 Feb 17;11(2):e0148546. doi: 10.1371/journal.pone.0148546 (PMC4757549; doi:10.1371/journal.pone.0148546)

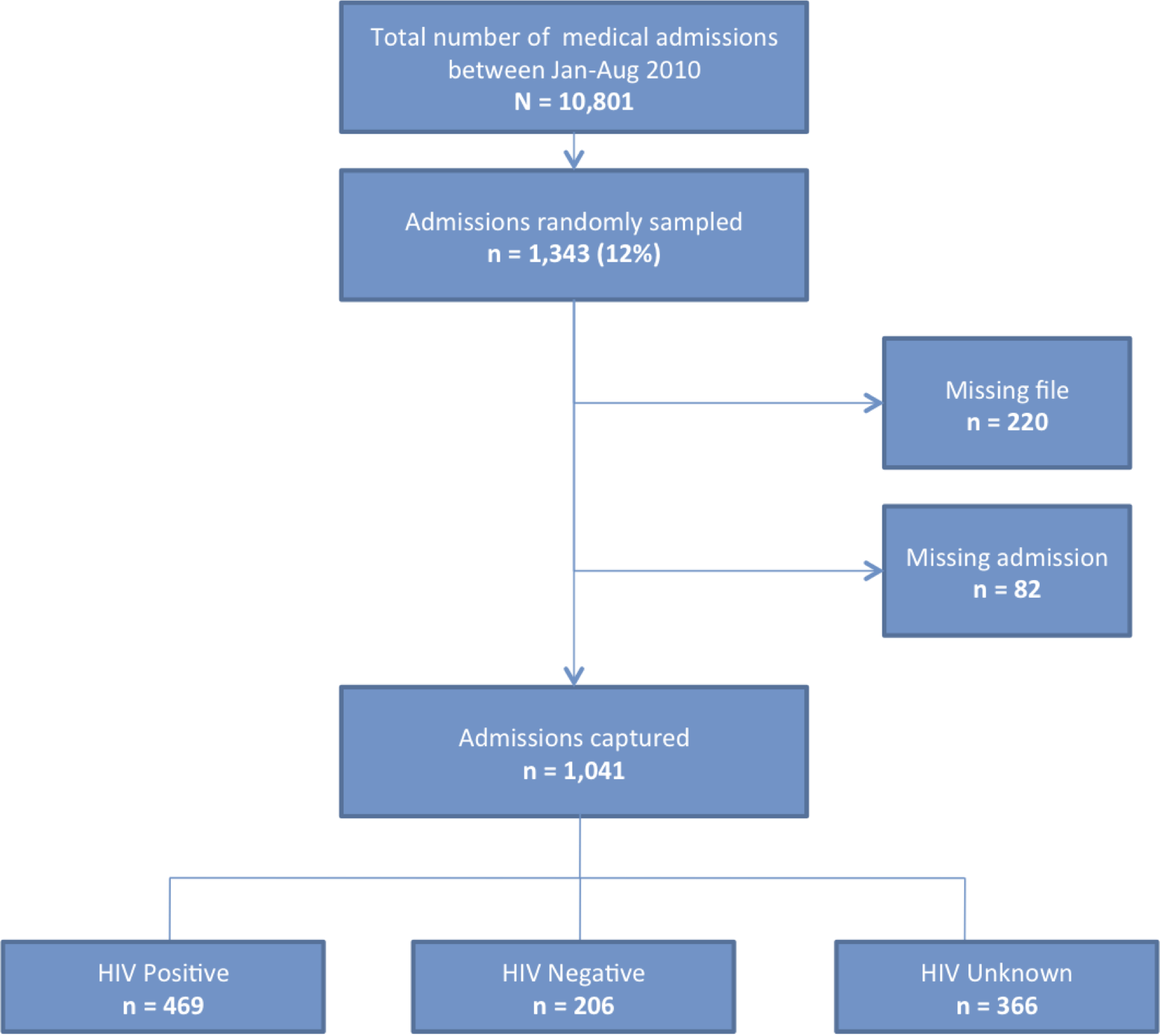

Supplement: S1 Fig — There were 10,801 medical admissions between January and August 2010. A sample of 10% was targeted with an additional 2% to account for missing or incomplete files. Of the 1,343 files selected 220 could not be found and an additional 82 could be identified but the admission of interest was missing or incomplete in the file. There were 1,041 admissions captured representing 9.6% of all admissions during the period. The majority of the admissions were confirmed as HIV-positive (466, 45%), while the rest were HIV-negative (206, 20%) and those with an unknown HIV status (366, 35%). (TIFF) [file pone.0148546.s001.tiff]
