# Supplementary material for: The High Cost of HIV-Positive Inpatient Care at an Urban Hospital in Johannesburg, South Africa
Source: PLoS One. 2016 Feb 17;11(2):e0148546. doi: 10.1371/journal.pone.0148546 (PMC4757549; doi:10.1371/journal.pone.0148546)
Supplement: S1 File — Table A. Reason of admission by HIV status. Table B. Summary of reasons for admission by HIV status. Table C. Primary reason for admission by ART status, HIV-positive only. Table D. Length of stay (days) by reason of admission and HIV status. Table E. Summary length of stay (days) by HIV status and reason for admission. Table F. Distribution of bed days by reason of admission. Table G. Mean cost per admission (HIV-positive only), by cost category in USD. (DOCX) [file pone.0148546.s002.docx]

**S1 TABLES**

**Table A.** Reason of admission by HIV status

| **Reason for admission** | **Overall** | | **HIV status** | | | | | | | |
| --- | --- | --- | --- | --- | --- | --- | --- | --- | --- | --- |
|  |  |  | **Positive** | | **Negative** | | | | **Unknown** | |
| **n (%)** |  |  |  |  |  |  |  |  |  |  |
| TB and other mycobacterial infections* | 187 | (18.0) | 164 | (35.0) | | 6 | (1.6) | 17 | | (8.3) |
| Cardiovascular disorder* | 127 | (12.2) | 20 | (4.3) | | 72 | (19.7) | 35 | | (17.0) |
| Bacterial infection* | 121 | (11.6) | 79 | (16.8) | | 22 | (6.0) | 20 | | (9.7) |
| Unknown* | 106 | (10.2) | 38 | (8.1) | | 43 | (11.7) | 25 | | (12.1) |
| Pulmonary disease* | 73 | (7.0) | 29 | (6.2) | | 21 | (5.7) | 23 | | (11.2) |
| Hypertension* | 56 | (5.4) | 1 | (0.2) | | 47 | (12.8) | 8 | | (3.9) |
| Nervous system disorder | 51 | (4.9) | 12 | (2.6) | | 23 | (6.3) | 16 | | (7.8) |
| Diabetes* | 45 | (4.3) | 3 | (0.6) | | 34 | (9.3) | 8 | | (3.9) |
| Psychiatric* | 39 | (3.7) | 8 | (1.7) | | 27 | (7.4) | 4 | | (1.9) |
| Diarrheal disease* | 38 | (3.7) | 29 | (6.2) | | 8 | (2.2) | 1 | | (0.5) |
| Hematologic disorder | 25 | (2.4) | 16 | (3.4) | | 6 | (1.6) | 3 | | (1.5) |
| Renal disease | 24 | (2.3) | 6 | (1.3) | | 8 | (2.2) | 10 | | (4.9) |
| Viral infections | 24 | (2.3) | 9 | (1.9) | | 6 | (1.6) | 9 | | (4.4) |
| Gastro-intestinal disease | 23 | (2.2) | 5 | (1.1) | | 15 | (4.1) | 3 | | (1.5) |
| Drug adverse event | 22 | (2.1) | 5 | (1.1) | | 13 | (3.6) | 4 | | (1.9) |
| Cancer | 21 | (2.0) | 10 | (2.1) | | 6 | (1.6) | 5 | | (2.4) |
| HIV and related OI | 21 | (2.0) | 21 | (4.5) | | 0 | 0 | 0 | | 0 |
| Parasitic infection | 12 | (1.2) | 5 | (1.1) | | 2 | (0.5) | 5 | | (2.4) |
| Musculo-skeletal | 9 | (0.9) | 2 | (0.4) | | 2 | (0.5) | 5 | | (2.4) |
| Endocrine and metabolic disease | 5 | (0.5) | 1 | (0.2) | | 2 | (0.5) | 2 | | (1.0) |
| Dermatologic condition | 4 | (0.4) | 2 | (0.4) | | 0 | 0 | 2 | | (1.0) |
| Other | 3 | (0.3) | 2 | (0.4) | | 0 | 0 | 1 | | (0.5) |
| Trauma | 3 | (0.3) | 0 | 0 | | 3 | (0.8) | 0 | | 0 |
| Obstetrics and gynecology | 2 | (0.2) | 2 | (0.4) | | 0 | 0 | 0 | | 0 |
| ***Total*** | ***1041*** | ***(100.0)*** | ***469*** | ***(100.0)*** | | ***366*** | ***(100.0)*** | ***206*** | | ***(100.0)*** |

* These were the top 5 unique reasons by HIV status and used in summary Table A-2

**Table B.** Summary of reasons for admission by HIV status

| **Reason for admission** | **Overall** | | **HIV status** | | | | | |
| --- | --- | --- | --- | --- | --- | --- | --- | --- |
|  |  |  | **Positive** | | **Negative** | | **Unknown** | |
| **n (%)** |  |  | **n=469** | | **n=206** | | **n= 366** | |
| TB and other mycobacterial infections | 187 | (18.0) | 164 | (35.0) | 17 | (8.3) | 6 | (1.6) |
| Cardiovascular disorder | 127 | (12.2) | 20 | (4.3) | 35 | (17.0) | 72 | (19.7) |
| Bacterial infection | 121 | (11.6) | 79 | (16.8) | 20 | (9.7) | 22 | (6.0) |
| Unknown | 106 | (10.2) | 38 | (8.1) | 25 | (12.1) | 43 | (11.7) |
| Pulmonary disease | 73 | (7.0) | 29 | (6.2) | 23 | (11.2) | 21 | (5.7) |
| Hypertension | 56 | (5.4) | 1 | (0.2) | 8 | (3.9) | 47 | (12.8) |
| Diabetes | 45 | (4.3) | 3 | (0.6) | 8 | (3.9) | 34 | (9.3) |
| Psychiatric | 39 | (3.7) | 8 | (1.7) | 4 | (1.9) | 27 | (7.4) |
| Diarrheal disease | 38 | (3.7) | 29 | (6.2) | 1 | (0.5) | 8 | (2.2) |
| Other* | 249 | (23.9) | 98 | (20.9) | 86 | (23.5) | 65 | (31.6) |
| ***Total*** | ***1041*** | ***(100.0)*** | ***469*** | ***(100.0)*** | ***206*** | ***(100.0)*** | ***366*** | ***(100.0)*** |

* Includes all other reasons which were not the amongst the top 5 most frequent reasons for any HIV status group

**Table C.** Primary reason for admission by ART status, HIV-positive only

| **Reason for admission** | **Overall** | | **ARV status** | | | | | |
| --- | --- | --- | --- | --- | --- | --- | --- | --- |
| **n (%)** |  |  | **Not on ARV** | | **On ARV** | | | |
| TB and other mycobacterial infections | 164 | (35.0) | 103 | (34.6) | | 61 | (35.7) |  |
| Bacterial infection | 79 | (16.8) | 63 | (21.1) | | 16 | (9.4) |  |
| Unknown | 38 | (8.1) | 26 | (8.7) | | 12 | (7.0) |  |
| Diarrheal disease | 29 | (6.2) | 17 | (5.7) | | 12 | (7.0) |  |
| Pulmonary disease | 29 | (6.2) | 15 | (5.0) | | 14 | (8.2) |  |
| Other* | 130 | (27.7) | 74 | (24.8) | | 56 | (32.7) |  |
| ***Total*** | **469** | **(100.0)** | **298** | **(100.0)** | | **171** | **(100.0)** |  |

* Includes all other reasons which were not the amongst the top 5 most frequent reasons

**Table D.** Length of stay (days) by reason of admission and HIV status

| **Reason for admission** | **Overall** | | **HIV status** | | | | | | | |
| --- | --- | --- | --- | --- | --- | --- | --- | --- | --- | --- |
|  |  |  | **Positive** | | **Negative** | | | | **Unknown** | |
| **n, mean** |  |  |  |  |  |  |  |  |  |  |
| HIV and related OI* | 21 | 13.2 | 21 | 13.2 | | . | . | . | | . |
| Cancer* | 21 | 12.5 | 10 | 8.2 | | 5 | 23.4 | 6 | | 10.7 |
| Renal disease* | 24 | 11.3 | 6 | 17.8 | | 10 | 10.0 | 8 | | 8.0 |
| Other* | 3 | 10.3 | 2 | 9.0 | | 1 | 13.0 | . | | . |
| TB and other mycobacterial infections* | 186 | 10.0 | 163 | 9.9 | | 17 | 11.8 | 6 | | 5.7 |
| Unknown | 81 | 8.5 | 29 | 12.3 | | 20 | 6.6 | 32 | | 6.3 |
| Viral infections* | 24 | 8.2 | 9 | 8.2 | | 9 | 10.3 | 6 | | 4.8 |
| Hematologic disorder | 25 | 7.8 | 16 | 9.3 | | 3 | 6.3 | 6 | | 4.7 |
| Cardiovascular disorder* | 127 | 7.4 | 20 | 9.9 | | 35 | 7.1 | 72 | | 6.8 |
| Bacterial infection | 121 | 7.3 | 79 | 7.9 | | 20 | 6.8 | 22 | | 5.7 |
| Gastro-intestinal disease* | 23 | 7.3 | 5 | 14.8 | | 3 | 4.0 | 15 | | 5.4 |
| Trauma* | 3 | 7.0 | . | . | | . | . | 3 | | 7.0 |
| Parasitic infection | 12 | 6.9 | 5 | 10.0 | | 5 | 4.2 | 2 | | 6.0 |
| Pulmonary disease | 73 | 6.6 | 29 | 7.1 | | 23 | 6.6 | 21 | | 5.8 |
| Dermatologic condition | 4 | 5.8 | 2 | 9.5 | | 2 | 2.0 | . | | . |
| Diabetes | 45 | 5.6 | 3 | 9.0 | | 8 | 5.8 | 34 | | 5.3 |
| Endocrine and metabolic disease* | 5 | 5.6 | 1 | 13.0 | | 2 | 4.5 | 2 | | 3.0 |
| Diarrheal disease | 38 | 5.4 | 29 | 6.0 | | 1 | 2.0 | 8 | | 3.5 |
| Nervous system disorder* | 51 | 5.4 | 12 | 3.3 | | 16 | 3.8 | 23 | | 7.5 |
| Drug adverse event* | 22 | 5.0 | 5 | 13.8 | | 4 | 4.5 | 13 | | 1.8 |
| Hypertension | 56 | 4.9 | 1 | 1.0 | | 8 | 5.9 | 47 | | 4.8 |
| Psychiatric | 39 | 4.6 | 8 | 7.1 | | 4 | 7.0 | 27 | | 3.5 |
| Musculo-skeletal | 9 | 4.2 | 2 | 11.5 | | 5 | 2.2 | 2 | | 2.0 |
| Obstetrics and gynecology | 2 | 1.0 | 2 | 1.0 | | . | . | . | | . |
| ***Total*** | ***1015*** | ***7.6*** | ***459*** | ***9.3*** | | ***201*** | ***7.3*** | ***355*** | | ***5.6*** |

* These were the top 5 unique reasons by HIV status and used in summary Table A-5

**Table E.** Summary length of stay (days) by HIV status and reason for admission

| **Reason** | **Overall** | | | | **Positive** | | **Negative** | | **Difference (Neg-Pos)** | | | **Unknown** | |
| --- | --- | --- | --- | --- | --- | --- | --- | --- | --- | --- | --- | --- | --- |
|  | **N** | **Mean** | **95% CI** | | **N** | **Mean** | **N** | **Mean** | **Mean** | **95% CI** | | **N** | **Mean** |
| HIV and related OI | 21 | 13.2 | (8.9, | 17.3) | 21 | 13.2 | - | - | - | - | - | - | - |
| Cancer | 21 | 12.5 | (7.4, | 17.6) | 10 | 8.2 | 5 | 23.4 | 15 | (-1.7, | 32.1) | 6 | 10.7 |
| Renal disease | 24 | 11.3 | (8.2, | 14.3) | 6 | 17.8 | 10 | 10.0 | -7.8 | (19.9, | 2.2) | 8 | 8.0 |
| TB and other mycobacterial infections | 186 | 10.0 | (8.8, | 11.0) | 163 | 9.9 | 17 | 11.8 | 1.9 | (-2.5, | 6.4) | 6 | 5.7 |
| Viral infections | 24 | 8.2 | (5.6, | 10.7) | 9 | 8.2 | 9 | 10.3 | 2.1 | (-5.8, | 10.1) | 6 | 4.8 |
| Cardiovascular disorder | 127 | 7.4 | (6.4, | 8.3) | 20 | 9.9 | 35 | 7.1 | -2.7 | (-7.3, | 1.9) | 72 | 6.8 |
| Gastro-intestinal disease | 23 | 7.3 | (4.2, | 10.2) | 5 | 14.8 | 3 | 4.0 | -10.8 | (-32.5, | 10.9) | 15 | 5.4 |
| Trauma | 3 | 7.0 | (5.1, | 19.1) | - | - | - | - | - | - | - | 3 | 7.0 |
| Endocrine and metabolic disease | 5 | 5.6 | (1.0, | 10.1) | 1 | 13.0 | 2 | 4.5 | -8.5 | (-63.5, | 46.5) | 2 | 3.0 |
| Nervous system disorder | 51 | 5.4 | (4.1, | 6.5) | 12 | 3.3 | 16 | 3.8 | 0.4 | (-2.1, | 3.1) | 23 | 7.5 |
| Drug adverse event | 22 | 5.0 | (2.7, | 7.3) | 5 | 13.8 | 4 | 4.5 | -9.3 | (-19.5, | 0.9) | 13 | 1.8 |
| Other* | 508 | 6.6 | (6.0, | 7.0) | 207 | 8.3 | 100 | 6.1 | -2.1 | (-3.9, | -0.3) | 201 | 5.1 |
| ***Total***** | ***1015*** | ***7.6*** | ***(7.2,*** | ***8.0)*** | ***459*** | ***9.3*** | ***201*** | ***7.3*** |  |  |  | ***355*** | ***5.6*** |

* Includes all other reasons which were not the amongst the top 5 most frequent reasons

** All admissions where the outcome is unknown are excluded, as they do not have a discharge date

**Table F.**  Distribution of bed days by reason of admission

| **Reason for admission** | **Overall** | | **HIV status** | | | | | | | |
| --- | --- | --- | --- | --- | --- | --- | --- | --- | --- | --- |
|  |  |  | **Positive** | | **Negative** | | | | **Unknown** | |
| **Total bed days (%)** |  |  |  |  |  |  |  |  |  |  |
| TB and other mycobacterial infections* | 1852 | (18.3) | 1617 | (35.5) | | 201 | (8.5) | 34 | | (1.7) |
| Cardiovascular disorder* | 936 | (12.5) | 197 | (4.4) | | 249 | (17.4) | 490 | | (20.3) |
| Bacterial infection* | 887 | (11.9) | 625 | (17.2) | | 136 | (10.0) | 126 | | (6.2) |
| Unknown* | 687 | (8.0) | 356 | (6.3) | | 131 | (10.0) | 200 | | (9.0) |
| Pulmonary disease* | 480 | (7.2) | 207 | (6.3) | | 151 | (11.4) | 122 | | (5.9) |
| HIV and related OI | 277 | (2.1) | 277 | (4.6) | | 0 | 0 | 0 | | 0 |
| Nervous system disorder | 273 | (5.0) | 40 | (2.6) | | 61 | (8.0) | 172 | | (6.5) |
| Hypertension* | 273 | (5.5) | 1 | (0.2) | | 47 | (4.0) | 225 | | (13.2) |
| Renal disease | 271 | (2.4) | 107 | (1.3) | | 100 | (5.0) | 64 | | (2.3) |
| Cancer | 263 | (2.1) | 82 | (2.2) | | 117 | (2.5) | 64 | | (1.7) |
| Diabetes* | 253 | (4.4) | 27 | (0.7) | | 46 | (4.0) | 180 | | (9.6) |
| Diarrheal disease* | 205 | (3.7) | 175 | (6.3) | | 2 | (0.5) | 28 | | (2.3) |
| Viral infections | 196 | (2.4) | 74 | (2.0) | | 93 | (4.5) | 29 | | (1.7) |
| Hematologic disorder | 196 | (2.5) | 149 | (3.5) | | 19 | (1.5) | 28 | | (1.7) |
| Psychiatric* | 180 | (3.8) | 57 | (1.7) | | 28 | (2.0) | 95 | | (7.6) |
| Gastro-intestinal disease | 167 | (2.3) | 74 | (1.1) | | 12 | (1.5) | 81 | | (4.2) |
| Drug adverse event | 111 | (2.2) | 69 | (1.1) | | 18 | (2.0) | 24 | | (3.7) |
| Parasitic infection | 83 | (1.2) | 50 | (1.1) | | 21 | (2.5) | 12 | | (0.6) |
| Musculo-skeletal | 38 | (0.9) | 23 | (0.4) | | 11 | (2.5) | 4 | | (0.6) |
| Other | 31 | (0.3) | 18 | (0.4) | | 13 | (0.5) | 0 | | 0 |
| Endocrine and metabolic disease | 28 | (0.5) | 13 | (0.2) | | 9 | (1.0) | 6 | | (0.6) |
| Dermatologic condition | 23 | (0.4) | 19 | (0.4) | | 4 | (1.0) | 0 | | 0 |
| Trauma | 21 | (0.3) | 0 | 0 | | 0 | 0 | 21 | | (0.8) |
| Obstetrics and gynecology | 2 | (0.2) | 2 | (0.4) | | 0 | 0 | 0 | | 0 |
| ***Total*** | ***7733*** | ***(100.0)*** | ***4259*** | ***(100.0)*** | | ***1469*** | ***(100.0)*** | ***2005*** | | ***(100.0)*** |

* These were the top 5 unique reasons by HIV status and used in summary Table 5

**Table G.** Mean cost per admission (HIV-positive only), by cost category in USD

| **Reason for Admission** | **N** | **Total** | **Fixed** | **Event** | **Lab** | **Drug+ARV** | **Fluid** |
| --- | --- | --- | --- | --- | --- | --- | --- |
| Renal disease | 6 | 3,944 | 1,241 | 1,459 | 372 | 110 | 762 |
| Drug adverse event | 5 | 2,576 | 961 | 1,129 | 234 | 21 | 232 |
| Endocrine and metabolic disease | 1 | 2,472 | 905 | 1,063 | 363 | 140 | 1 |
| HIV and related OI | 21 | 2,435 | 918 | 1,079 | 218 | 83 | 137 |
| Gastro-intestinal disease | 5 | 2,394 | 1,030 | 1,211 | 132 | 20 | 2 |
| Unknown | 29 | 2,350 | 652 | 766 | 219 | 69 | 198 |
| Hematologic disorder | 16 | 2,038 | 648 | 762 | 241 | 52 | 336 |
| Parasitic infection | 5 | 1,927 | 696 | 818 | 189 | 98 | 126 |
| TB and other mycobacterial infections | 163 | 1,922 | 686 | 806 | 198 | 66 | 165 |
| Musculo-skeletal | 2 | 1,880 | 800 | 941 | 93 | 44 | 3 |
| Other | 2 | 1,807 | 626 | 736 | 196 | 59 | 189 |
| Cardiovascular disorder | 20 | 1,784 | 686 | 806 | 223 | 32 | 37 |
| Viral infections | 9 | 1,783 | 572 | 673 | 167 | 120 | 250 |
| Cancer | 10 | 1,621 | 571 | 671 | 176 | 63 | 140 |
| Diabetes | 3 | 1,586 | 626 | 736 | 174 | 40 | 9 |
| Dermatologic condition | 2 | 1,580 | 661 | 777 | 137 | 4 | 1 |
| Bacterial infection | 79 | 1,470 | 551 | 647 | 147 | 71 | 53 |
| Psychiatric | 8 | 1,413 | 496 | 583 | 212 | 15 | 107 |
| Pulmonary disease | 29 | 1,355 | 497 | 584 | 173 | 52 | 49 |
| Diarrheal disease | 29 | 1,151 | 420 | 494 | 147 | 21 | 69 |
| Nervous system disorder | 12 | 683 | 232 | 273 | 118 | 23 | 37 |
| Hypertension | 1 | 282 | 70 | 82 | 131 | 0 | - |
| Obstetrics and gynecology | 2 | 280 | 70 | 82 | 125 | 3 | 1 |
| ***All*** | ***459*** | ***1,783*** | ***632*** | ***743*** | ***188*** | ***61*** | ***134*** |
